# Diffuse large B-cell lymphoma with concurrent high MYC and BCL2 expression shows evidence of active B-cell receptor signaling by quantitative immunofluorescence

**DOI:** 10.1371/journal.pone.0172364

**Published:** 2017-02-17

**Authors:** Agata M. Bogusz, Alexandra E. Kovach, Long P. Le, Derek Feng, Richard H. G. Baxter, Aliyah R. Sohani

**Affiliations:** 1 Department of Pathology and Laboratory Medicine, University of Pennsylvania, Philadelphia, Pennsylvania, United States of America; 2 Department of Pathology, Microbiology and Immunology, Vanderbilt University Medical Center, Nashville, Tennessee, United States of America; 3 Department of Pathology, Massachusetts General Hospital, Boston, Massachusetts, United States of America; 4 Department of Statistics, Yale University, New Haven, Connecticut, United States of America; 5 Department of Chemistry, Yale University, New Haven, Connecticut, United States of America; 6 Department of Molecular Biophysics & Biochemistry, Yale University, New Haven, Connecticut, United States of America; Cornell University, UNITED STATES

## Abstract

B-cell receptor (BCR)-mediated signaling plays an important role in the pathogenesis of a subset of diffuse large B-cell lymphoma (DLBCL), and novel agents targeting this pathway are now in clinical use. We have previously identified a signature of active BCR signaling on formalin-fixed paraffin-embedded specimens using quantitative immunofluorescence, allowing for identification of patients who might benefit from anti-BCR therapies. We sought to characterize the clinicopathologic significance of active BCR signaling in DLBCL by correlating measures of signaling intensity with clinical features and various tumor cell characteristics. High MYC and concurrent high MYC and BCL2 double-expression was positively correlated with individual markers of active BCR signaling and cases with MYC/BCL2 double-expression showed overall greater BCR activation compared to cases lacking double-expression. Our findings suggest that the BCR signaling pathway may be more active in MYC/BCL2 double-expressor DLBCL and may represent a rational therapeutic target in this aggressive DLBCL subgroup.

## Introduction

Diffuse large B-cell lymphoma (DLBCL) is the most common type of non-Hodgkin lymphoma and has a poor prognosis in approximately 50% of patients [1,2]. DLBCL is biologically heterogeneous and shows variable responses to conventional chemotherapy and rituximab [1,2]. Recent advances in next generation sequencing (NGS) have provided a better understanding of the biology of DLBCL and have implicated potential targets to improve diagnosis and therapy [3–5]. A variety of signaling pathways are involved in the pathogenesis of DLBCL including those involving the B-cell receptor (BCR), NFκB, NOTCH, Toll-like receptor (TLR), PI3 kinase, MAP kinase, immunity, cell cycle/apoptosis, and chromatin modification [3,5–7]. A number of recurrent mutations lead, either directly or indirectly, to pathway activation, and components of various signaling pathways are attractive targets in the clinical setting [3].

Based on cell-of-origin (COO) gene signatures studies, DLBCL can be stratified into prognostically relevant subtypes, with the activated B-cell (ABC) type being associated with an inferior outcome compared with the germinal center B-cell (GCB) type [8–10]. With regard to BCR signaling inhibition, fostamatinib, a SYK inhibitor, showed activity against relapsed DLBCL in an early phase I/II study [11], and evaluable tumor responses were observed in two of seven DLBCL patients treated with the BTK inhibitor, ibrutinib, as part of a phase I study for relapsed or refractory B-cell non-Hodgkin lymphoma [12]. In a subsequent phase II study of fostamatinib, the overall response rate was only 3% and no patient with stable disease or response had ABC type tumors [13]. However, ibrutinib continues to hold promise as an effective strategy for BCR signal inhibition, at least in certain DLBCL subtypes: in a recent phase I/II clinical trial of relapsed or refractory DLBCL, ibrutinib achieved 37% complete or partial responses in ABC subtype but only 5% response in GCB DLBCL, underscoring the importance of BCR signaling in the pathogenesis of ABC type tumors [14]. Interestingly, this study showed that the highest number of responses occurred in ABC tumors that lacked BCR mutations (9/29; 31%) implying that BCR signaling in ABC DLBCL is not dependent on the presence of BCR mutations and may be activated via other mechanisms [14]. Genetic and transcriptional analyses are not yet widely available in routine practice, and although paraffin-based methods are on the horizon [15], these analyses represent only an indirect measure of protein expression. Immunohistochemistry (IHC) is a time-tested and widely available method to evaluate both protein amount and phosphorylation. Thus, immunohistochemical analysis of BCR signaling components should be an effective and accurate tool for selection of DLBCL cases that would respond to anti-BCR therapies, such as ibrutinib.

We have previously identified a robust signature of active BCR signaling in DLBCL on formalin-fixed paraffin-embedded specimens based on quantitative immunofluorescence (qIF) of phosphorylated BCR-associated kinases SYK, LYN and BTK [16]. We used DLBCL cell lines as a model system, and identified and validated active BCR signaling in 46% (71/154) of primary DLBCL patient specimens in two clinical cohorts. Additional analysis revealed increased nuclear exclusion of forkhead transcription factor FOXO1, a downstream effect of BCR signaling and AKT activation leading to increased cell survival, among DLBCL with qIF evidence of active BCR signaling compared with those without (*p* = 0.004). There was no difference between any qIF variable for GCB versus non-GCB cases, nor was there any enrichment for GCB or non-GCB COO within BCR-positive or negative cases [16]. Our data underscored the importance of immunohistochemical analysis to detect active BCR signaling at the level of protein expression and supported the utility of qIF as a tool to identify patients who could potentially benefit from anti-BCR therapies such as ibrutinib.

Rearrangements of the oncogenes *BCL6*, *BCL2*, as well as *MYC* are well documented in DLBCL, and *MYC* translocations are recognized to confer a worse prognosis in patients treated with cyclophosphamide, doxorubicin, vincristine, and prednisone (CHOP), with or without rituximab (R) [17–20]. The poor outcome of double-hit DLBCL, defined by rearrangements affecting the *MYC* locus in combination with another breakpoint, mainly *BCL2*, appears to be the result of the combination of MYC and BCL2 overexpression [21–23]. While double-hit DLBCL is relatively uncommon, found in 5–10% of cases, concurrent high expression of MYC and BCL2 proteins by IHC, termed double-expressor lymphoma (DEL), is detected in approximately 20% of DLBCL [22,24,25]. DEL is characterized by an aggressive clinical course and inferior response to R-CHOP therapy, indicating the need for more individualized therapeutic approaches targeting particular signaling pathways [23–30]. MYC overexpression by IHC does not correlate perfectly with the presence of a *MYC* rearrangement, as other mechanisms of *MYC* activation can lead to overexpression, including copy number amplification, upstream regulation by microRNA and oncogenic mutations. However, *MYC* gene activation has been associated with poor outcome and thus, these alternate pathways that activate *MYC* may have similar biological consequences as *MYC* rearrangement. Indeed, some studies have shown that B-cell lymphomas with concurrent *MYC* and *BCL2* abnormalities, other than translocations, appear to behave similarly to *MYC/BCL2* double-hit lymphomas [27–29]. In addition, it has been proposed that the poor prognosis of ABC vs. GCB type in DLBCL may be largely explained by MYC/BCL2 double-expression rather than COO per se [26], although COO assignment using an RNA-based expression platform designed for formalin-fixed paraffin-embedded tissue appears to support the prognostic impact of COO independent of MYC/BCL2 double-expression [31]. Currently, these IHC studies are increasingly used as part of risk stratification of DLBCL but do not directly impact therapy [25,26]. In this study, we further characterize the clinicopathological significance of active BCR signaling, as determined by qIF, in a cohort of primary DLBCL samples (*N* = 93) by correlating signaling intensity with clinical features and tumor cell characteristics. We demonstrate that concurrent high expression of MYC and BCL2 by IHC is positively correlated with previously studied markers of activated BCR signaling. These findings imply that the BCR signaling pathway is more active in DEL compared to other DLBCL subgroups and may represent a rational therapeutic target in this aggressive subgroup of DLBCL.

## Methods

### Case selection

To analyze clinicopathologic features associated with activated BCR signaling in a cohort of primary DLBCL samples, we obtained clinical follow-up data from electronic medical records of patients from the original validation cohort from the previously published study [16]. This cohort consisted of 144 consecutive DLBCL patients diagnosed at the Massachusetts General Hospital between 2000 and 2006. Patients were identified via a computer-assisted search of electronic pathology reports and only cases with sufficient tissue for tissue microarray (TMA) construction were selected for further study following Institutional Review Board approval. Tumors were classified according to the 2008 World Health Organization classification [1].

### Tissue microarray construction, immunofluorescence and immunohistochemistry

TMA construction was conducted as described previously [32]. Briefly, three 0.6 mm diameter tissue cores were punched from representative regions of each donor tissue block and inserted into a recipient paraffin block using a semiautomatic robotic precision instrument. Immunofluorescent staining and quantification on the TMA was performed as described previously [16] and as detailed in S1 Text. Tumors were further characterized by IHC for various antigens previously shown to be prognostic in DLBCL. TMA sections 4 μm thick were prepared, deparaffinized and rehydrated according to laboratory protocols. Staining was performed using Leica BOND Polymer Refine DAB Detection kits on a Leica BOND-III Autostainer (Leica Biosystems, Buffalo Grove, IL) using validated staining protocols. On line antigen retrieval was performed prior to incubation with the primary antibodies. Antibodies studied, and their clones, dilutions and sources were as follows: MYC (clone Y69, 1:50, Epitomics), BCL2 (clone bcl-2/100/D5, prediluted, Leica), CD10 (clone 56C6, prediluted, Leica), BCL6 (clone LN22, prediluted, Leica), MUM1 (clone EAU32, prediluted, Leica), Ki67 (clone MIB-1, 1:200, Dako), CD30 (clone BerH2, 1:50, Dako) and p53 (clone DO-7, prediluted; Leica).

All immunohistochemical stains were evaluated via consensus review by two hematopathologists (AEK and ARS), who were blinded to the results of qIF and to patient outcome. Stains for MYC, BCL2, Ki67, CD30 and p53 were scored on tumor cells in increments of 10%. Based on prior data examining optimum survival cutoffs for dichotomizing levels of expression, 40% positivity was used as a cut-off for MYC and 50% positivity for BCL2 overexpression [25]. Therefore, DEL was defined as tumors demonstrating ≥40% MYC and ≥50% BCL2 expression. COO (GCB vs. non-GCB) was determined using the Hans classifier (CD10, BCL6, MUM1), with positive expression for each antibody defined as ≥30% tumor cell staining [33].

### Fluorescence In Situ Hybridization (FISH)

FISH was performed on 4μm thick TMA sections as described previously [34]. Rearrangements involving the following loci were assessed using the probes indicated (each from Abbot Molecular, Des Plaines, IL): *MYC*/8q24 (Vysis LSI *MYC* Dual Color Break-Apart Rearrangement Probe), *BCL2*/18q21 (Vysis LSI *BCL2* Dual Color Break-Apart Rearrangement Probe), and *BCL6*/3q27 (Vysis LSI *BCL6* Dual Color Break-Apart Rearrangement Probe). Fifty to 100 nuclei were scored per case, and a case was considered positive for the rearrangement if 20% or more nuclei exhibited a break-apart signal.

### Statistical analysis

The initial statistical analysis of qIF data has been previously described [16]. The mean and standard deviation of pLYN+(%), combined score of pSYK+(%) and pBTK+(%) expression derived from linear regression using a maximum-likelihood algorithm (〈pSYK,pBTK〉), and cytoplasmic localization score of FOXO1 (F_cyt_), a surrogate marker of AKT activation, were calculated. Results from untransformed data are based upon arithmetic means, and transformed data are based upon medians, of two to three samples per clinical specimen. Details regarding transformation and reanalysis of BCR signaling classification are provided in S1 Text. Welch's unequal variances *t*-test was used to compare qIF markers (pLYN, pSYK, pBTK, FOXO1), adjusted where noted by the Holm-Bonferroni correction. Pearson’s χ^2^ test with Yates’ correction was used to compare nominal variables. Overall survival (OS) was defined as the time from diagnosis to death from any cause. OS was analyzed using the Kaplan-Meier method with censoring. The log-rank test used to compare differences in survival between groups.

## Results

**Fig 1:**
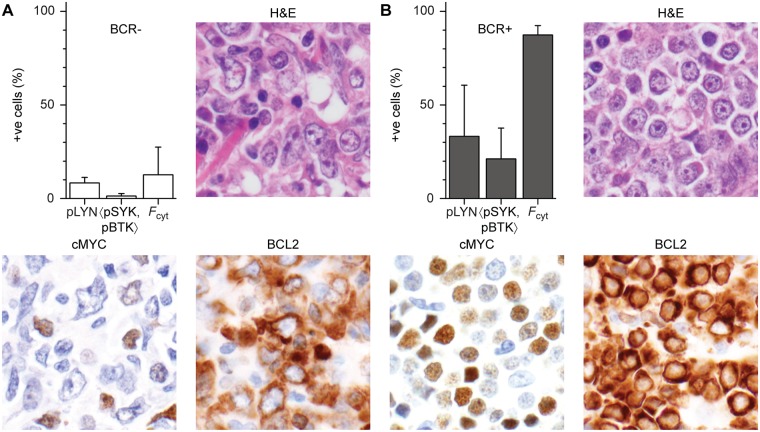
Differences in MYC and BCL2 expression by IHC in a BCR-negative and a BCR-positive case. Bar graph of percent positive (+ve) for BCR phosphomarkers pLYN, <pSYK, pBTK> and *F*_cyt_ and representative images of H&E stain and MYC and BCL2 immunohistochemical stains for two representative cases. (A) A BCR-negative case that was negative for MYC and positive for BCL2 expression. This patient responded to R-CHOP therapy and was alive with no evidence of disease 10 years following diagnosis. (B) A BCR-positive case with MYC/BCL2 double-expression. This patient expired within several months of diagnosis despite receiving R-CHOP.

Of 144 clinical specimens examined, 93 fulfilled criteria for measurement of pLYN, 〈pSYK,pBTK〉 and *F*_cyt_ and were evaluable for BCR signaling by qIF. Among the immunohistochemical markers studied, high MYC expression (27/93 cases, 29%) and DEL (21/93 cases, 23%) were found to be positively correlated with markers of activated BCR signaling by qIF (Table 1). The significance of the association increased progressively for markers further downstream of the BCR, from pLYN (*p* = 0.08), to 〈pSYK,pBTK〉 (*p* = 0.02), to *F*_cyt_ (*p* = 4×10^−5^). Examples of BCR+ and BCR- cases and their differences in MYC and BCL2 expression are illustrated in Fig 1.

**Table 1:** Clinicopathologic and qIF characteristics of DEL vs. non-DEL.

Category	All	MYC+^1^	MYC-^1^	DEL^2^	non-DEL^2^	MYC+ vs. MYC-^3^	DEL vs. non-DEL^3^
*N*	93	27	66	21	72		
Clinical parameters							
Age^4^	62±16	64±16	61±16	66±14	61±16	ns	ns
Male	57	19	38	15	42		
Female	36	8	28	6	30	ns	ns
Treatment							
CHOP	16	5	11	5	11		
R-CHOP	56	14	42	11	45		
R-other	6	2	4	2	4		
Other	5	1	4	1	4		
Unknown	10	5	5	2	8		
Immunophenotype							
pLYN^+^ (%)^4^	36±32	51±35	30±28	48±35	33±30	0.008	0.08
〈pSYK,pBTK〉 (%)^4^	29±29	44±30	24±27	44±32	25±27	0.005	0.02
*F*_cyt_^4^	52±30	70±22	45±30	73±21	47±30	4×10^−5^	4×10^−5^
GCB	50	10	40	8	42		
non-GCB	43	17	26	13	30	ns	ns
BCL2 (%)^4^	45±37	69±36	36±34	86±16	34±34	2×10^−4^	6×10^−15^
CD30 (%)^4^	8±18	4±13	10±19	4±13	10±19	ns	ns
Ki67 (%)^4^	63±24	73±23	59±23	69±23	62±23	0.009	ns
p53 (%)^4^	19±28	29±33	14±25	27±30	16±23	0.05	ns
FISH (+ve/-ve/ND)^5^							
*MYC*	7/81/5	5/19/3	2/62/2	4/14/3	3/67/2	ns	ns
*BCL2*	13/79/1	3/23/1	10/56/0	3/17/1	10/62/0	ns	ns
*BCL6*	8/76/9	3/20/4	5/56/5	2/15/4	6/61/5	ns	ns

^1^ MYC ≥ 40%.

^2^ MYC ≥ 40%, BCL2 ≥ 50%.

^3^ *p*-values are uncorrected for multiple comparisons. Holm-Bonferroni corrections (MYC+ vs. MYC-): *p* = 0.02 (pLYN), *p* = 0.02 (〈pSYK,pBTK〉), *p* = 2×10^−4^ (*F*_cyt_), *p* = 0.001 (BCL2), *p* = 0.02 (Ki67), *p* = 0.05 (p53). Holm-Bonferroni corrections (DEL vs. non-DEL): *p* = 0.08 (pLYN), *p* = 0.04 (〈pSYK,pBTK〉), *p* = 1×10^−4^ (*F*_cyt_), *p* = 2×10^−14^ (BCL2).

^4^ Expressed as mean +/- standard deviation.

^5^ No cases of *MYC/BCL2* or *MYC/BCL6* double-hit lymphoma were identified.

Abbreviations: CHOP—cyclophosphamide, doxorubicin, vincristine, and prednisone; COO—cell of origin; DEL—double-expressor lymphoma; FISH—fluorescence in situ hybridization; GCG—germinal center B-cell type; IHC—immunohistochemistry; ns—not significant; R-CHOP—rituximab, cyclophosphamide, doxorubicin, vincristine, and prednisone; R-other—rituximab alone or other rituximab-containing regimen; qIF—quantitative immunofluorescence.

No correlation was observed between BCR signaling markers and the other clinical or pathological parameters studied, including age; sex; COO as assessed by the Hans classifier; expression of BCL2 alone, BCL6, Ki67, CD30 or p53 by IHC; or *MYC*, *BCL2* or *BCL6* rearrangement status as determined by FISH. Of note, only 8% of cases harbored a *MYC* rearrangement and no cases of *MYC/BCL2* or *MYC/BCL6* double-hit lymphoma were identified.

Based on previously established criteria [16], we initially classified cases with >15% pLYN and >15% 〈pSYK,pBTK〉 as BCR-positive (BCR+), and cases with <15% pLYN and <15% 〈pSYK,pBTK〉 as BCR-negative (BCR-). Despite the observed association of BCR signaling markers with DEL, we found no significant association of DEL with classification as BCR+, although there was a significant association of DEL with *F*_cyt_ (Table 2). We therefore re-examined our criteria for BCR classification. Two problems are evident: first, the use of percentage positive cells is not optimal because the data is not evenly distributed over the range of 0–100%; and second, the definition of BCR+ and BCR- excludes cases with >15% pLYN but <15% 〈pSYK,pBTK〉, or *vice versa*, corresponding to 18/93 (19%) of cases in the current cohort.

**Table 2:** Association of DEL with BCR and *F*_cyt_ classification (untransformed).

	DEL	non-DEL	Pearson’s χ^2^	*p* value
All cases	21	72		
BCR+	13	29	3.75	0.15
BCR-	4	29		
Other*	4	14		
*F*_cyt_+ (>50)	17	30	10.04	0.001
*F*_cyt_- (<50)	4	42		

*Cases with >15% pLYN but <15% 〈pSYK,pBTK〉 or *vice versa* that are unclassified based on previously established criteria for BCR positivity.^16^

Abbreviations: BCR—B-cell receptor; DEL—double-expressor lymphoma.

Accordingly, we returned to the original qIF data for BCR signaling markers in activated DLBCL cell lines [16] and applied a modified logit transformation to correct the uneven distribution of data (S1 Fig). Unsupervised normal mixture modeling readily clustered known BCR+ and BCR- cell lines (S2 Fig). We then defined a hyperplane equally weighted according to the three transformed BCR markers, pLYN, pSYK and pBTK, in order to separate the two clusters for unambiguous assignment of all specimens as either BCR+ or BCR- (S1 Text).

**Fig 2:**
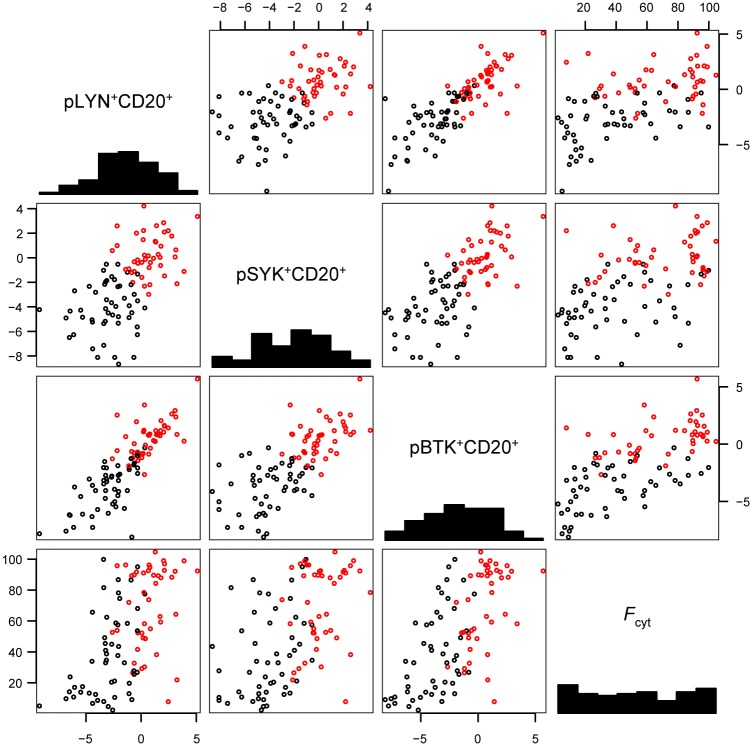
Logistic transform of BCR signaling markers in DLBCL specimens. Pairwise scatterplots of transformed (logit) data for pLYN^+^CD20^+^, pSYK^+^CD20^+^, pBTK^+^CD20^+^ and the untransformed ratio of FOXO1 cytoplasmic staining (*F*_cyt_) in 93 primary DLBCL cases. Cases are classified as BCR+ (red) or BCR- (black) based on a hyperplane based upon normal mixture modeling of BCR activation in ten DLBCL cell lines (S1 Text).

We applied the same transformation to the 93 clinical samples of DLBCL. The untransformed data were unevenly distributed, being clustered near the origin and 100% (S3 Fig). The logit transformed data were more evenly distributed (Fig 2), in closer agreement with a standard error distribution assumed by a standard *t*-test. Following logit transformation, DEL cases showed significantly higher levels for each individual BCR signaling marker compared to non-DEL cases, including after Holm-Bonferroni correction for multiple hypotheses testing (Table 3).

**Table 3:** Association of DEL with BCR classification according to logit transformation.

	DEL	Non-DEL	*t*-test*
pLYN	0.027	-1.59	*p* = 0.02 (0.02)
pSYK	-0.66	-2.36	*p* = 4×10^−3^ (8×10^−3^)
pBTK	0.025	-2.21	*p* = 2×10^−3^ (5×10^−3^)
*F*_cyt_	70	47	*p* = 7×10^−4^ (3×10^−3^)

*p values in parentheses adjusted for Holm-Bonferroni correction.

Abbreviations: BCR—B-cell receptor; DEL—double-expressor lymphoma.

We tested recursive partitioning to generate a decision tree (S4 Fig), but this was not an effective means for selecting DEL cases on the basis of BCR signaling markers. We also applied unsupervised clustering by normal mixture modeling to the qIF data of the TMA (S1 Text). Two clusters were generated for putative BCR+ and BCR- cases, but no significant association between BCR status and DEL was detected. However, when the hyperplane derived from reanalysis of DLBCL cell lines was applied to classify BCR+ and BCR- cases, a significant association was observed between BCR status and both DEL and *F*_cyt_ via this revised classification (Table 4).

**Table 4:** Association of DEL with BCR (transformed) and of BCR (transformed) with *F*_cyt_ classification.

	DEL	Non-DEL	Pearson’s χ^2^	*p* value
BCR+	15	28	5.68	0.02
BCR-	6	44		
	**BCR+**	**BCR-**		
*F*_cyt_+ (>50)	33	15	18.40	2×10^−5^
*F*_cyt_- (<50)	10	35		

Abbreviations: BCR—B-cell receptor; DEL—double-expressor lymphoma.

**Fig 3:**
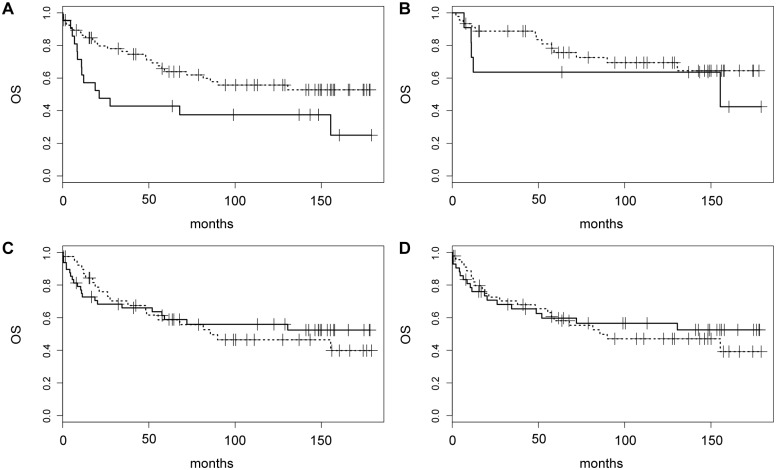
Kaplan-Meier analysis of overall survival (OS) based on DEL and BCR classification. (A) OS for DEL (solid) vs. non-DEL (dashed) cases (*p* = 0.03) among all patients with available follow-up. (B) OS for DEL (solid) vs. non-DEL (dashed) cases restricted to R-CHOP-treated patients. (C) OS for BCR+ (solid) vs. BCR- (dashed) cases according to the revised definition for BCR positivity. (D) OS for cases with *F*_cyt_>50 (solid) vs. *F*_cyt_<50 (dashed).

Treatment information was known in 83/93 patients (89%), the largest proportion of whom received R-CHOP (56/83, 67%) (Table 1). Survival data were available in 88/93 patients (95%), with a median follow-up of 60 months. Patients with DEL had significantly shorter OS compared to those with non-DEL (median OS 21 months vs. not reached, *p* = 0.03, Fig 3A). The difference between DEL and non-DEL was no longer significant when analysis was restricted to R-CHOP-treated patients, likely due to the smaller sample size (Fig 3B). In addition, no significant OS difference was seen between BCR+ vs. BCR- cases (Fig 3C), or between cases with net cytoplasmic localization of FOXO1 (*F*_cyt_>50 vs. F_cyt_<50, Fig 3D). There were also no statistically significant differences in OS between DEL/BCR-, DEL/BCR+, non-DEL/BCR- and non-DEL/BCR+ cases (S5 Fig). Hence, while active BCR signaling and FOXO1 cytoplasmic localization were associated with DEL, they showed no apparent association with OS in the cohort comprising this study.

## Discussion

In this study, we demonstrate that among several pathological features assessed by IHC and FISH, MYC expression by IHC was the only one positively correlated with markers of active BCR signaling as determined by qIF. In addition, DEL showed greater BCR activation as compared to non-DEL based on individual phosphomarker expression, FOXO1 cytoplasmic localization, and overall BCR activity classification. Overall, our findings suggest that the BCR signaling pathway is more active in DEL compared with other DLBCL subgroups and raise the possibility for targeting the BCR signaling pathway with agents, such as ibrutinib, in this inferior prognostic subgroup of DLBCL.

Although DEL correlated with both OS and activated BCR signaling, we were unable to demonstrate a direct association between active BCR signaling and OS, which may be related to the retrospective nature of our study, the relatively small number of patients within different immunohistochemical subgroups, and the non-uniform treatment received (as treatment was not a selection criterion for inclusion in our TMA), all important limitations of our analysis. However, our findings suggest that study of a larger number of uniformly treated patients may help to clarify the prognostic and predictive implications, if any, of activated BCR signaling in DLBCL and DEL. In addition, as with immunohistochemical studies of a similar nature, pre-analytical variables and inter- and intraobserver variability in scoring tissue sections may confound the results. Therefore, validation of these findings is warranted following the development of multiplex quantitative IHC assays for simultaneous and objective scoring of multiple markers.

The exact mechanisms linking BCR signaling to MYC expression have yet to be elucidated. The *MYC* oncogene is a transcription factor that has a broad effect on gene expression: it regulates more than 15% of human genes, including a number of microRNA (miRNAs), and contributes to the pathogenesis of many human cancers [35,36]. Activation of MYC has a profound role in a variety of cellular processes including proliferation, DNA replication, metabolism, and protein and nucleotide biosynthesis. Hence, its dysregulation is associated with genomic instability and oncogenic potential. Studies of P493-6, a human B-lymphoid cell line with tet-repressible *MYC* gene and enrichment of *MYC*-repressed genes, show evidence for the role of miR-17~92, a MYC-regulated miRNA, as a major regulator of BCR pathway components [37]. The miR-17~92 cluster has a strong oncogenic role: it is known to regulate multiple cellular processes that contribute to malignant transformation, cell survival and rapid cell proliferation, and it has been implicated in various B-cell malignancies, including DLBCL [38–41]. In the P493-6 cell line, many of the direct targets of miR-17~92 were found to be immunoreceptor tyrosine inhibitory motif (ITIM)-containing proteins, including CD22 [37]. Interestingly, either MYC or miR-17~92 expression was necessary to sustain phosphorylation of SYK and B-cell linker protein (BLNK) upon BCR activation. Furthermore, BCR stimulation by miR-17~92 resulted in elevated MYC protein levels and enhanced calcium influx, while inhibition of the miR-17~92 diminished the BCR response as measured by SYK and BLNK phosphorylation. Thus, it appears that the MYC-miR-17~92-BCR axis may constitute a novel lymphomagenic feed-forward loop in which MYC amplifies BCR signaling and increases its own protein levels via upregulation of miR-17~92 and targeting of ITIM proteins. This study also showed that human DLBCLs of the BCR subtype by molecular profiling express higher levels of *MYC* transcript and *MIR17HG*, the precursor transcript derived from the *miR-17~92* gene, compared to other subtypes [42]. Other data suggest that MYC may act as a universal amplifier of expressed genes in lymphocytes and embryonic stem cells by enhancing preexisting transcriptional programs rather than being an on/off specifier [43,44]. Therefore, it is possible that our results simply reflect increased BCR-mediated gene transcription that is present to some extent in all DLBCL but further regulated and enhanced by overexpression of MYC. A recent study in precursor B-cell acute lymphoblastic leukemia revealed that pre-BCR signaling regulates PI3K/AKT, FOXO1 and MYC and can be a target of SYK inhibition [45]. Interestingly, this report shows that the pre-BCR regulates MYC in a FOXO1-dependent manner. Finally, early data show that MYC and BCR signaling may also be interconnected through regulation of MYC stability via post-translational modification and phosphorylation at specific MYC residues [46].

DEL is characterized by an aggressive clinical course and inferior response to R-CHOP therapy, indicating the need for more individualized therapeutic approaches targeting particular signaling pathways [23–26]. Active BCR signaling can be detected using qIF of phosphorylated forms of BCR-associated kinases LYN, SYK and BTK in nearly 50% of DLBCL and can be used as a tool in formalin-fixed paraffin-embedded tissue samples to identify patients who may benefit from anti-BCR therapies [16,47,48]. Our findings suggest that the BCR signaling pathway as assessed by qIF shows higher activity in MYC-high DLBCL and DEL compared with other DLBCL subgroups. We also confirm the utility of MYC and BCL2 IHC, tests that are more readily available and less costly than FISH, to identify DLBCL cases with an unfavorable prognosis. Our preliminary findings suggesting the potential utility of BCR signaling inhibitors in DEL require validation in prospective studies incorporating MYC/BCL2 double-staining and qIF of BCR signaling molecules to identify patients most likely to respond to such agents. Such a strategy may also help to clarify the interplay between BCR signaling and MYC overexpression in DLBCL.

## Supporting information

S1 Text Supporting methods. (PDF)

S1 Fig Logistic transform as a function of percentage. Plot of logit(*p*) vs. *p*.
(TIF)

S2 Fig Logistic transform and cluster analysis of BCR signaling markers in DLBCL cell lines. (A) Pairwise scatterplots of untransformed data for %pLYN+, %pSYK+, %pBTK+ in ten DLBCL cell lines. Uneven distribution with crowding at the origin is evident. (B) Pairwise scatterplots of transformed (logit) data for %pLYN+, %pSYK+, %pBTK+. (C) Pairwise scatterplots of transformed (logit) data for %pLYN+, %pSYK+, %pBTK+, with centroids for two clusters generated by unsupervised normal mixture modeling. BCR+ (blue), BCR- (red). (D) Pairwise scatterplots of aggregated (median) transformed data for %pLYN+, %pSYK+, %pBTK+, with centroids for two clusters generated by unsupervised normal mixture modeling. BCR+ (blue), BCR- (red).
(TIF)

S3 Fig Untransformed data for BCR signaling markers in primary DLBCL specimens. Pairwise scatterplots of untransformed data for %pLYN+CD20+, %pSYK+CD20+, %pBTK+CD20+ in tissue microarray of primary DLBCL specimens. Uneven distribution with crowding at the origin and near 100% is evident.
(TIF)

S4 Fig Recursive partitioning of DEL vs. non-DEL cases according to BCR signaling markers. Decision tree based on unsupervised recursive partitioning of DLBCL primary specimens according to BCR signaling markers. Each intersection within the tree is labeled based on the majority of cases, and the number of DEL cases (left) and non-DEL cases (right) given below and the percent (%) of all cases within the cohort. Below the tree are one- and two-dimensional scatterplots of cases based on single or pairwise classification by the four BCR signaling markers pLYN, pSYK, pBTK and Fcyt. Cases are assigned as either DEL (red) or non-DEL (blue), and cut-offs determined by recursive partitioning indicated by step function (1-D) or black/white masking (2D). Note: logistic transform for generation of plots used ε = 1×10–3, resulting in slight reduction in overall data range compared to other Figs.
(TIF)

S5 Fig Kaplan-Meier analysis of overall survival (OS) based on DEL and BCR classification. OS for DEL/BCR- (solid black), OS for DEL/BCR+ (dashed black line), non-DEL/BCR- (red solid) and non-DEL/BCR+ (dashed red).
(TIF)
