# Supplementary material for: Diffuse large B-cell lymphoma with concurrent high MYC and BCL2 expression shows evidence of active B-cell receptor signaling by quantitative immunofluorescence
Source: PLoS One. 2017 Feb 17;12(2):e0172364. doi: 10.1371/journal.pone.0172364 (PMC5315400; doi:10.1371/journal.pone.0172364)
Supplement: S1 Text — (PDF) [file pone.0172364.s001.pdf]

Supporting Methods

Quantitative immunofluorescence analysis of B-cell receptor (BCR) phosphomarkers and FOXO1 localization

To prepare the tissue microarray (TMA) for quantitative immunofluorescence (qIF) analysis, 4 µm slides were baked, soaked in xylene, passed through graded alcohols, and then pretreated with either Dako pH 9 retrieval solution (Dako, Carpinteria, CA) for pLYN, pSYK, and pBTK or 1 mmol/L EDTA at pH 8 (Zymed, Grand Island, NY) for FOXO1 in a steam pressure cooker (Decloaking Chamber; BioCare Medical, Concord, CA) as per manufacturer's instructions followed by washing in distilled water. All further steps were done at room temperature in a hydrated chamber. Slides were then treated with peroxidase block (Dako) for 5 minutes to quench endogenous peroxidase activity. Double-staining was conducted with 1:600 anti-pY396 LYN, 1:100 anti-pY323 SYK, 1:25 anti-pY551 BTK (Epitomics, Burlingame, CA) or 1:100 anti-FOXO1 and CD20 antibody (clone L26; Dako, ready to use solution) for 1 hour. After a wash in 50 mmol/L Tris-HCl (pH 7.4), the slides were labeled with 1:200 Alexa 555 goat anti-rabbit (phosphomarkers, FOXO1) and Texas Red-conjugated goat anti-mouse (CD20; Molecular Probes, Grand Island, NY) antibodies for 30 minutes and coverslipped using ProlongGold antifade with 4′, 6-diamidino-2-phenylindole (DAPI; Molecular Probes).

To quantify the qIF staining, the fluorescently double-stained (pan-B-cell marker CD20, along with either pLYN, pSYK, pBTK, or FOXO1) TMA slides were scanned using digital TissueFAXS imaging system and evaluated using TissueQuest imaging analysis software (TissueGnostics, Tarzana, CA). Single cells were identified using an algorithm based on a preselected so-called master channel (based on nuclear DAPI staining), and the intensity of staining within either a cytoplasmic or total cell mask was quantified. Positive cells were selected based on the cutoff values that were set by engaging negative controls and the backward and forward gating tool of the software.

The mean intensity of a phospho-marker/FOXO1 staining was plotted against mean intensity of CD20 to determine the absolute percentage of all cells that were double-positive, e.g.%pLYN^{+}CD20^{+}, %pSYK^{+}CD20^{+}, %pBTK^{+}CD20^{+}. To quantify the fraction of cells with cytoplasmic localization of FOXO1 the ratio of absolute double-positive cells was obtained for cells analyzed with a cytoplasmic mask vs. a whole-cell mask. Since TissueGnostics redraws cell masks in each case, the ratio was adjusted according to the absolute ratio of CD20 which were generally very similar, i.e.:

$$F_{\text{cyt}} = \frac{[\text{FOXO1}]_{\text{cyt}}}{[\text{CD20}]_{\text{cyt}}} \cdot \frac{[\text{CD20}]_{\text{tot}}}{[\text{FOXO1}]_{\text{tot}}} \times 100$$

Reanalysis of BCR classification of DLBCL cell lines

We reanalyzed qIF data for the three markers, pLYN, pSYK and pBTK, for a panel of 10 DLBCL cell lines – LY7, U2932, DHL4, DHL6, HBL-1, LY3, Pfeiffer, Toledo, K422 and LY4 – either untreated or stimulated by cross-linking surface immunoglobulin (sIg) with anti-immunoglobulin antibodies before fixation, in three separate experiments. Each data point consists of three percentages p in the range 0–100, but the data is unevenly distributed with untreated/negative values clustered near the origin and crosslinked, BCR active values varying widely. A standard mathematical operation that corrects for this is a modified logit function, commonly used in logistic regression [1-4],

$$\text{logit}(p) = \ln(\varepsilon + p/100) - \ln(1 + \varepsilon - p/100)$$

where p is the percent positive cells for a given marker, and ε a small positive value to provide a definite interval in the case p = 0, 100. The value $\varepsilon = 1\times10^{-4}$ was used for this study. A plot of

$\mathrm{logit}(p)$ vs. p is shown in Fig. S1. Compared to the raw data for the DLBCL cell lines (Fig. S2A), the logit-transformed data is more evenly distributed; the positive correlation between the variables is evident (Fig. S2B).

We then applied unsupervised normal mixture modeling to cluster the cell lines into BCR+ and BCR- specimens (Fig. S2C). The procedure correctly classified the cell lines previously identified as BCR+: LY7, U2932, DHL4, DHL6 and HBL-1. Similarly, the cell lines previously identified as BCR- were confirmed: Toledo, K422 and LY4. In contrast, unsupervised clustering of untransformed data incorrectly assigned 2/3 of K422 specimens as BCR+. Aggregating the data by selecting the median value for each cell line enhanced separation between the two clusters (Fig. S2D).

The cell lines LY3 and Pfeiffer were ambiguous when comparing BCR signaling assessed by qIF to RNA molecular profiling using a consensus clustering (CC) approach [5]. LY3 showed little activation of pSYK and pBTK despite being classified as a BCR cell line by CC molecular profiling. Conversely, Pfeiffer showed significant activation of all three markers despite being classified as BCR- (OxPhos). Clustering of the transformed data assigned 1/3 LY3 specimens as BCR+ and 2/3 Pfeiffer specimens as BCR- (Fig. S2C), but with significant uncertainty calculated for the classification of individual samples. Following aggregation (Fig. S2D), LY3 was assigned as BCR- while Pfeiffer was assigned as BCR+ in agreement with the majority classification of individual specimens, but with significant uncertainty in the classification of LY3.

In order to unambiguously assign unknown specimens as either BCR+ or BCR- based on the two clusters, a hyperplane can be defined between the two clusters. Samples above the plane are classified BCR+, and samples on or below the plane are classified BCR-. However, the limited number of cell lines in the training data is insufficient to determine with confidence the relative weight of each variable, i.e. the angle of the plane with respect to the three axes $\mathrm{logit(pLYN)}$, $\mathrm{logit(pSYK)}$ and $\mathrm{logit(pBTK)}$. We therefore adopted the null hypothesis that all three markers be given equal weight, yielding the following criterion for classification of samples as BCR+:

$$\mathrm{logit(pLYN)} + \mathrm{logit(pSYK)} + \mathrm{logit(pBTK)} > 4$$

This corresponds to a hyperbolic surface in untransformed space. For reference, the surface delineating BCR+ cases corresponds to the following percentages:

a) ≥0.5% of two markers, given one equal to 99.9%
b) ≥1.3% of two markers, given one equal to 99%
c) ≥11% of two markers, given one equal to 50%
d) ≥21% of all three markers
e) ≥29% of two markers, given one equal to 10%
f) ≥49% of two markers, given one equal to 2%
g) ≥58% of two markers, given one equal to 1%.
h) ≥81% of two markers, given one equal to 0.1%.

Finally, we examined the pairwise scatterplot of actual patient data from qIF analysis of the TMA (Fig. S3). The data is unevenly distributed as observed for the DLBCL cell lines, including clustering of data near 100% in some cases. This supports the value of applying the new transformation and classification to this cohort.

Recursive partitioning of transformed TMA data

A recursive partitioning algorithm was applied to generate a decision tree for selecting double-expressor lymphoma (DEL) cases and rejecting non-DEL cases on the basis of BCR signaling markers (Figure S4). Intriguingly, the order of decisions proceeds in approximate reverse order

to the signaling cascade, with BTK and FOXO1 above SYK, and LYN last of all. Selection of cases on very high BTK phosphorylation – *logit*(pBTK) ≥ 1.8 or > 85% – selected 9% (8/93) of the cohort with 75% (6/8) DEL cases, a 3-fold enrichment compared to all cases. Subsequent rejection of cases with predominantly nuclear FOXO1 ($F_{cyt} < 33$) and very low SYK phosphorylation – *logit*(pSYK) < -4.4 or < 1.1% – removed 41% (39/93) of all cases that were non-DEL, selecting 49% (46/93) of the cohort with 32% (15/46) DEL cases, a 50% enrichment compared to old cases. However, the remaining marker pLYN was not very effective at segregating DEL from non-DEL cases. The unsupervised partitioning arbitrarily selects a negative criterion – *logit*(pLYN) ≤ -1.2 or ≤ 23% – to select 17% (16/93) of the cohort with 56% (9/16) DEL cases, but rejects six DEL cases in the process. In summary, with the possible exception of F_{cyt} which rejects 33% (31/93) of the cohort that are all non-DEL cases, discrimination based on single BCR markers is not an effective means of selecting DEL cases.

References:

1. Andersen M, Grauslund M, Ravn J, Sorensen JB, Andersen CB, Santoni-Rugiu E. Diagnostic potential of miR-126, miR-143, miR-145, and miR-652 in malignant pleural mesothelioma. J Mol Diagn 2014;16:418-430.

2. Noh H, Park MS, Kim SH, et al. . Optimization of radotinib doses for the treatment of Asian patients with chronic myelogenous leukemia based on dose-response relationship analyses. Leuk Lymphoma 2015:1-9.

3. Van den Eynden GG, Colpaert CG, Vermeulen PB, et al. . Comparative analysis of the biochemical and immunohistochemical determination of hormone receptors in invasive breast carcinoma influence of the tumor-stroma ratio. Pathol Res Pract 2002;198:517-524.

4. Zhou Z, Chen ZW, Yang XH, et al. . Establishment of a biomarker model for predicting bone metastasis in resected stage III non-small cell lung cancer. J Exp Clin Cancer Res 2012;31:34.

5. Monti S, Savage KJ, Kutok JL, et al. . Molecular profiling of diffuse large B-cell lymphoma identifies robust subtypes including one characterized by host inflammatory response. Blood 2005;105:1851-1861.
