# Supplementary figures and images for: Diffuse large B-cell lymphoma with concurrent high MYC and BCL2 expression shows evidence of active B-cell receptor signaling by quantitative immunofluorescence
Source: PLoS One. 2017 Feb 17;12(2):e0172364. doi: 10.1371/journal.pone.0172364 (PMC5315400; doi:10.1371/journal.pone.0172364)

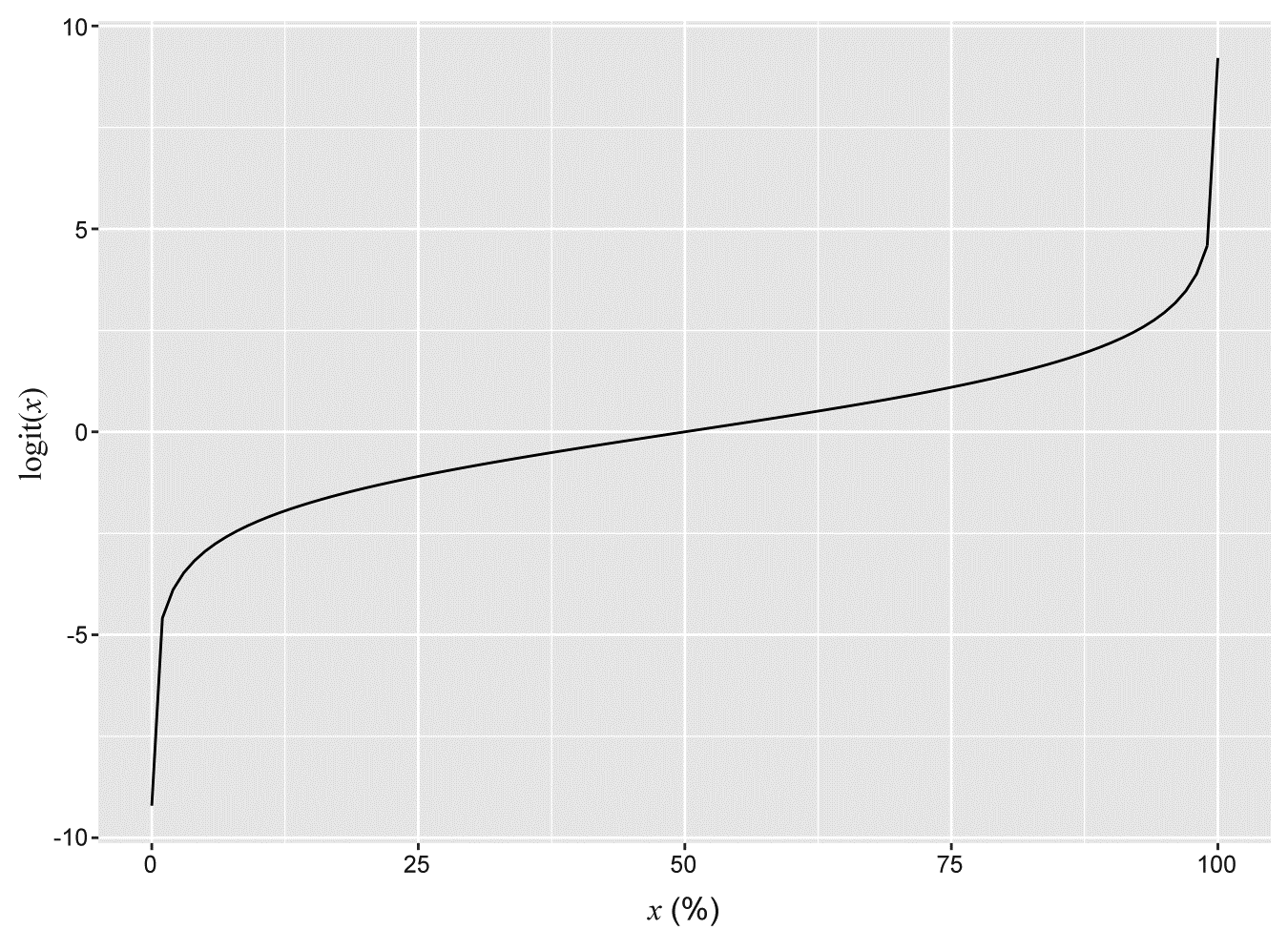

Supplement: S1 Fig — Plot of logit(p) vs. p. (TIF) [file pone.0172364.s002.tif]

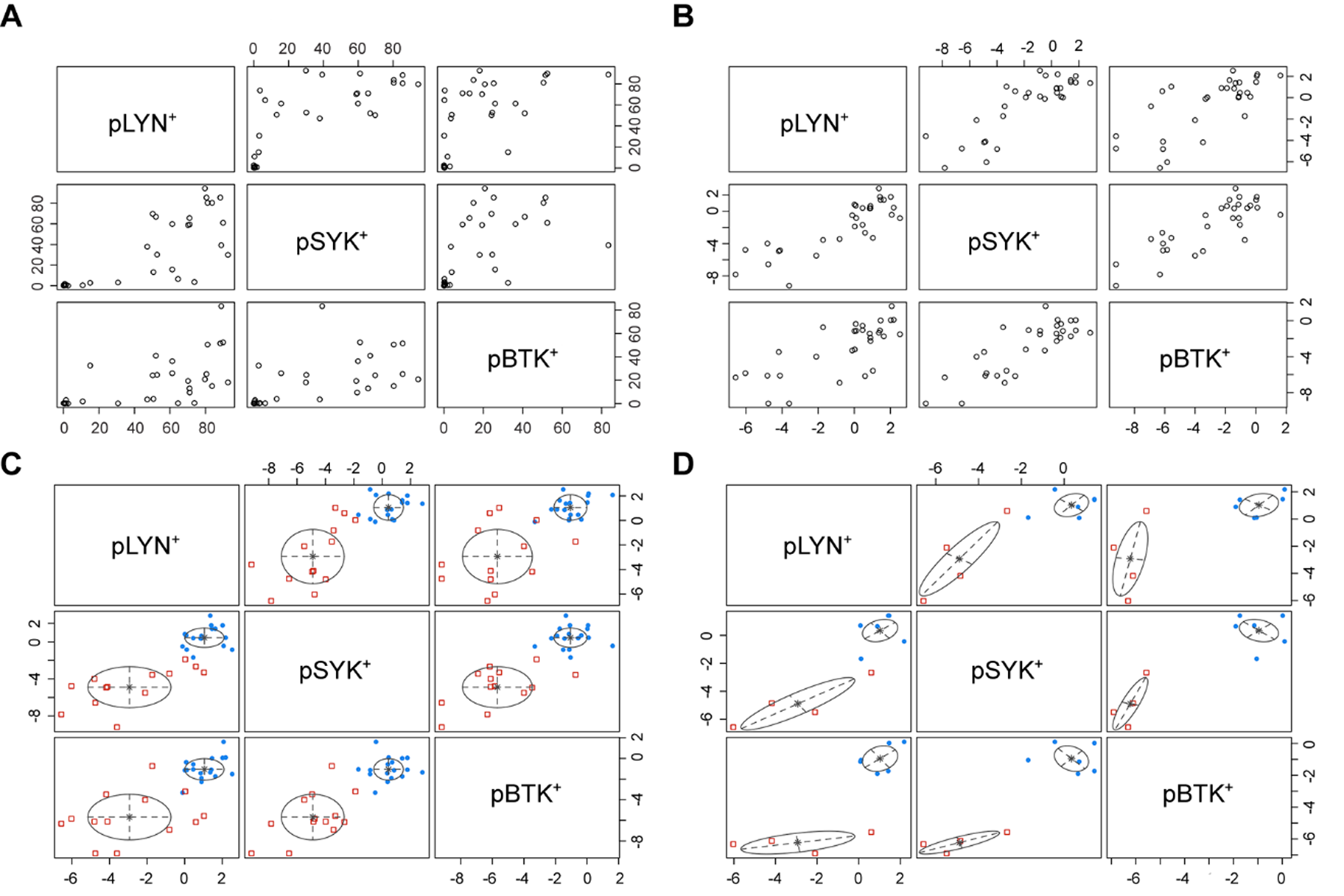

Supplement: S2 Fig — (A) Pairwise scatterplots of untransformed data for %pLYN+, %pSYK+, %pBTK+ in ten DLBCL cell lines. Uneven distribution with crowding at the origin is evident. (B) Pairwise scatterplots of transformed (logit) data for %pLYN+, %pSYK+, %pBTK+. (C) Pairwise scatterplots of transformed (logit) data for %pLYN+, %pSYK+, %pBTK+, with centroids for two clusters generated by unsupervised normal mixture modeling. BCR+ (blue), BCR- (red). (D) Pairwise scatterplots of aggregated (median) transformed data for %pLYN+, %pSYK+, %pBTK+, with centroids for two clusters generated by unsupervised normal mixture modeling. BCR+ (blue), BCR- (red). (TIF) [file pone.0172364.s003.tif]

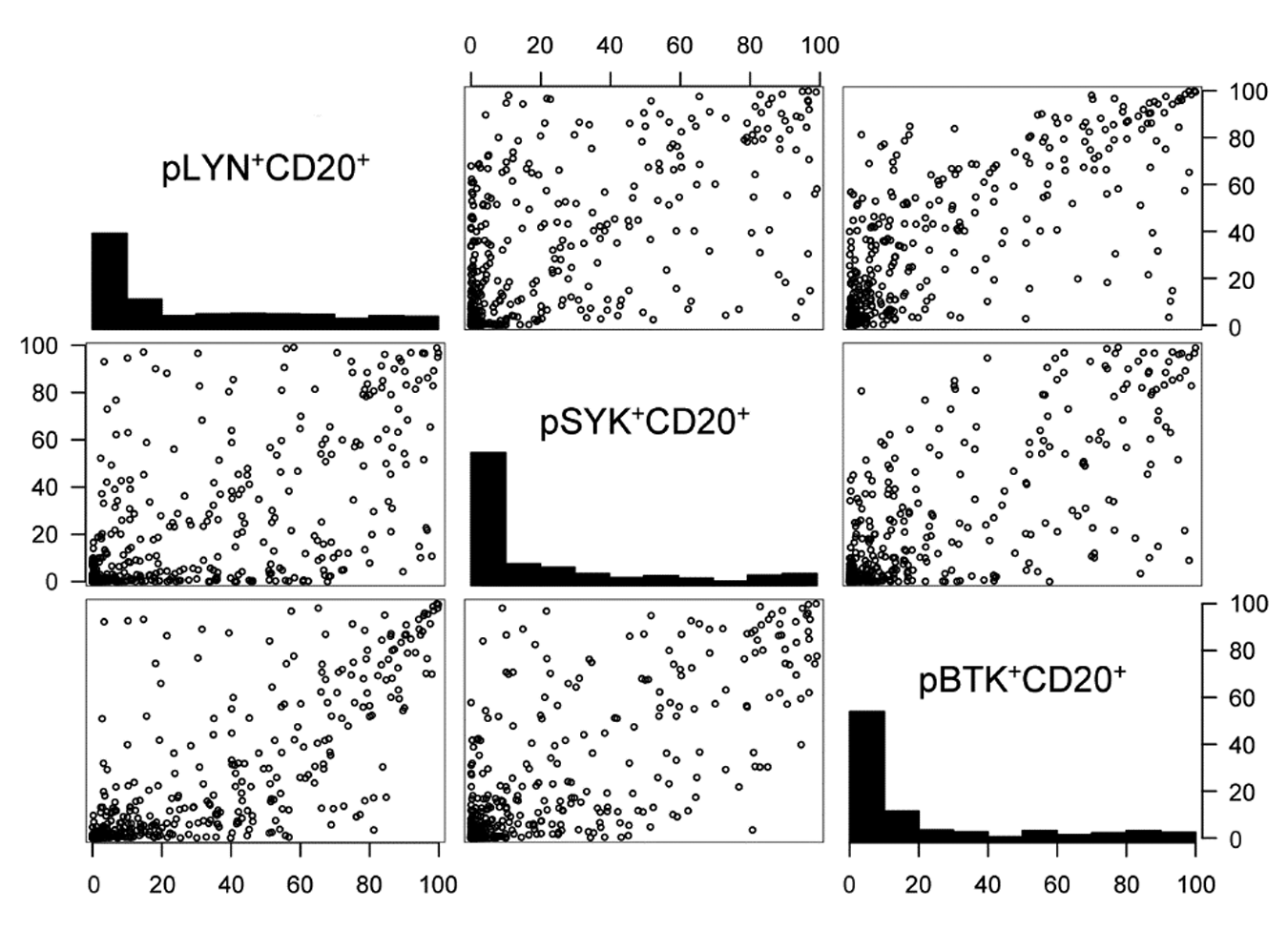

Supplement: S3 Fig — Pairwise scatterplots of untransformed data for %pLYN+CD20+, %pSYK+CD20+, %pBTK+CD20+ in tissue microarray of primary DLBCL specimens. Uneven distribution with crowding at the origin and near 100% is evident. (TIF) [file pone.0172364.s004.tif]

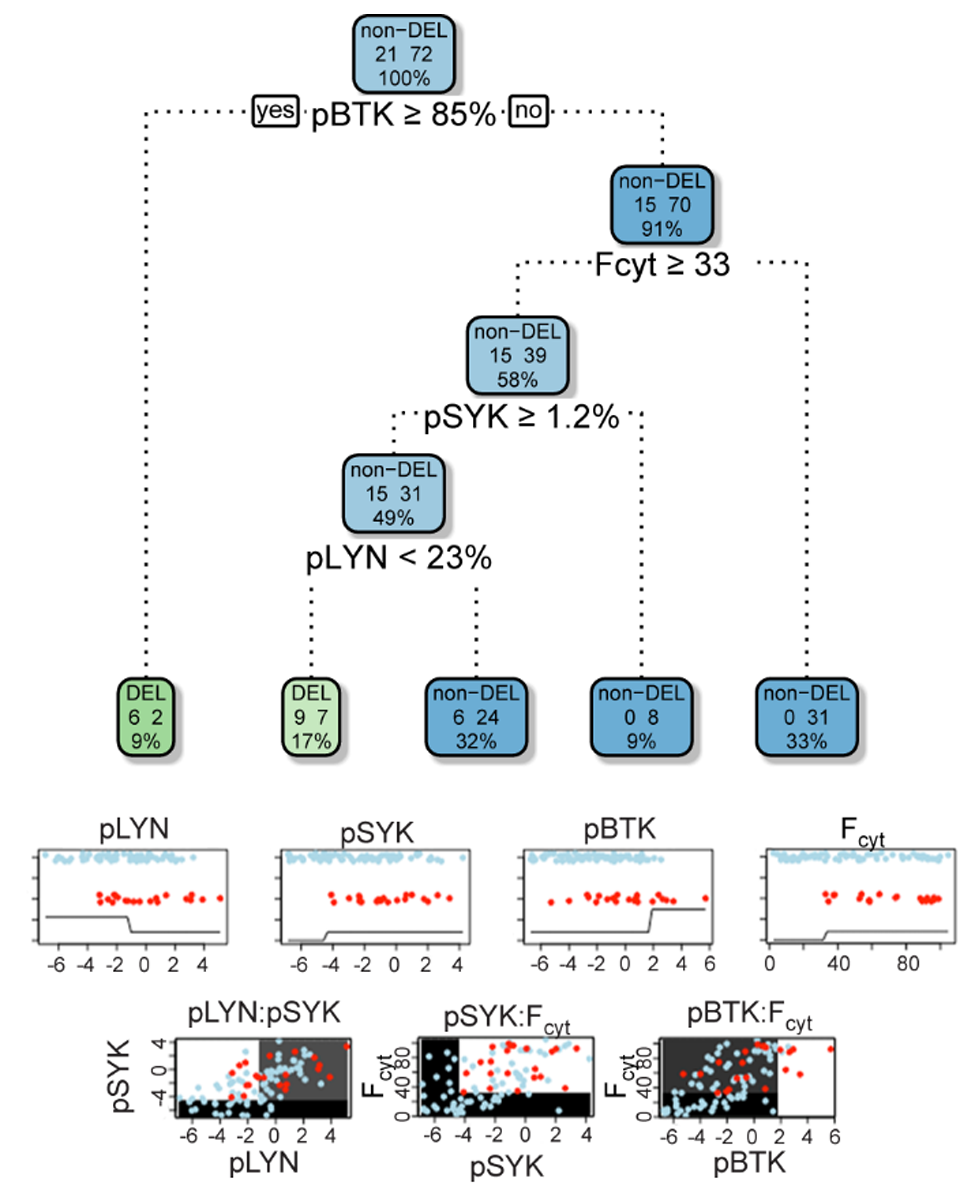

Supplement: S4 Fig — Decision tree based on unsupervised recursive partitioning of DLBCL primary specimens according to BCR signaling markers. Each intersection within the tree is labeled based on the majority of cases, and the number of DEL cases (left) and non-DEL cases (right) given below and the percent (%) of all cases within the cohort. Below the tree are one- and two-dimensional scatterplots of cases based on single or pairwise classification by the four BCR signaling markers pLYN, pSYK, pBTK and Fcyt. Cases are assigned as either DEL (red) or non-DEL (blue), and cut-offs determined by recursive partitioning indicated by step function (1-D) or black/white masking (2D). Note: logistic transform for generation of plots used ε = 1×10^–3^, resulting in slight reduction in overall data range compared to other Figs. (TIF) [file pone.0172364.s005.tif]

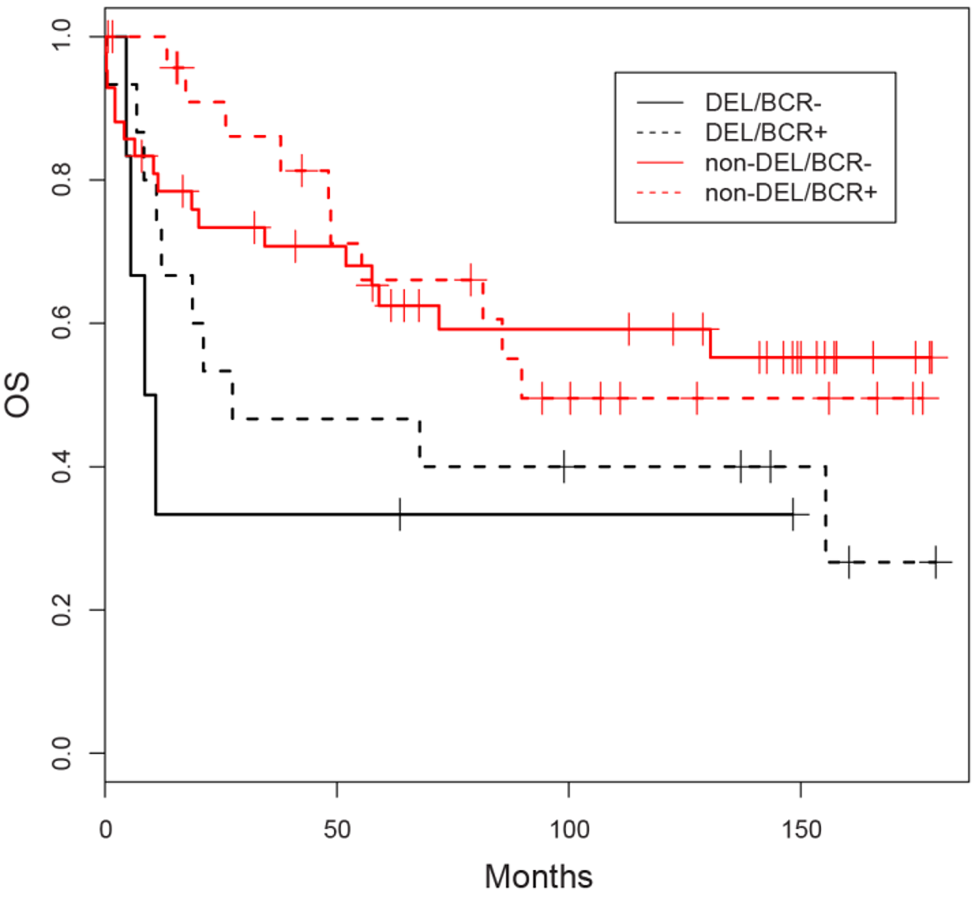

Supplement: S5 Fig — OS for DEL/BCR- (solid black), OS for DEL/BCR+ (dashed black line), non-DEL/BCR- (red solid) and non-DEL/BCR+ (dashed red). (TIF) [file pone.0172364.s006.tif]
